# Ventral tegmental area connections to motor and sensory cortical fields in humans

**DOI:** 10.1007/s00429-019-01939-0

**Published:** 2019-08-22

**Authors:** Jonas A. Hosp, V. A. Coenen, M. Rijntjes, K. Egger, H. Urbach, C. Weiller, M. Reisert

**Affiliations:** 1 Department of Neurology and Neuroscience, Freiburg University Medical Center, Breisacher Str. 64, 79106 Freiburg, Germany; 2 Department of Stereotactic and Functional Neurosurgery, Freiburg University Medical Center, Freiburg, Germany; 3 Department of Neuroradiology, Freiburg University Medical Center, Freiburg, Germany; 4 Department of Medical Physics, Freiburg University Medical Center, Freiburg, Germany; 5 Faculty of Medicine, University of Freiburg, Freiburg, Germany; 6 Center for Basics in Neuromodulation, University of Freiburg, Freiburg, Germany

**Keywords:** Brain, Dopamine, Medial forebrain bundle, Motor cortex, VTA

## Abstract

In humans, sensorimotor cortical areas receive relevant dopaminergic innervation—although an anatomic description of the underlying fiber projections is lacking so far. In general, dopaminergic projections towards the cortex originate within the ventral tegmental area (VTA) and are organized in a meso-cortico-limbic system. Using a DTI-based global tractography approach, we recently characterized the superolateral branch of the medial forebrain bundle (slMFB), a prominent pathway providing dopaminergic (and other transmitters) innervation for the pre-frontal cortex (Coenen et al., NeuroImage Clin 18:770–783, 2018). To define the connections between VTA and sensory–motor cortical fields that should contain dopaminergic fibers, we use the slMFB as a key structure to lead our fiber selection procedure: using a similar tracking-seed and tractography algorithm, we describe a dorsal extension of this slMFB that covers sensorimotor fields that are dorsally appended to pre-frontal cortical areas. This “motorMFB”, that connects the VTA to sensorimotor cortical fields, can be further segregated into three sub-bundles with a seed-based fiber-selection strategy: A PFC bundle that is attendant to the pre-frontal cortex, passes the lateral VTA, runs through the border zone between the posterior and lateral ventral thalamic nucleus, and involves the pre- and postcentral gyrus. An MB bundle that is attendant to the mammillary bodies runs directly through the medial VTA, passes the lateral ventral thalamic nucleus, and involves the pre- and postcentral gyrus as well as the supplementary motor area (SMA) and the dorsal premotor cortex (dPMC). Finally, a BC bundle that is attendant to the brainstem and cerebellum runs through the lateral VTA, passes the anterior ventral thalamic nucleus, and covers the SMA, pre-SMA, and the dPMC. We, furthermore, included a fiber tracking of the well-defined dentato-rubro-thalamic tract (DRT) that is known to lie in close proximity with respect to fiber orientation and projection areas. As expected, the tract is characterized by a decussation at the ponto-mesencephal level and a projection covering the superior-frontal and precentral cortex. In addition to the physiological role of these particular bundles, the physiological and pathophysiological impact of dopaminergic signaling within sensorimotor cortical fields becomes discussed. However, some limitations have to be taken into account in consequence of the method: the transmitter content, the directionality, and the occurrence of interposed synaptic contacts cannot be specified.

## Introduction

Although the functional role of cortical dopaminergic innervation is well defined for (limbic) medial frontal (Pierce and Kumaresan 2006) and pre-frontal (Puig et al. 2014) areas, growing evidence highlights the relevance of dopaminergic signaling within primary and secondary motor fields. For example, dopaminergic projections to the primary motor cortex are essential for successful motor learning in rodents (Hosp et al. 2011a, b) as they promote learning-related neuroplasticity (Hosp and Luft 2013). Conversely, dopaminergic denervation of motor cortical fields is thought to alter cortical excitability and to induce motor-learning impairments in patients suffering from Parkinson’s disease (Lindenbach and Bishop 2013). Furthermore, lower concentrations of dopamine within secondary motor areas have been linked to motor abnormalities in schizophrenia (Abboud et al. 2017).

Within the human cortex, dopaminergic fibers and terminals have been detected within primary motor (i.e., Brodmann area 4), secondary motor (i.e., Brodmann area 6), and somatosensory cortical areas (Gaspar et al. 1989; Sutoo et al. 2001; Raghanti et al. 2008). These dopaminergic fibers are part of the meso-cortical or meso-cortico-limbic pathway, a projection system that originates in the ventral tegmental area (VTA) and the neighboring medial substantia nigra (SN; Björklund and Dunnett 2007). This meso-cortico-limbic system provides dopaminergic input to both limbic “areas” (e.g., nucleus accumbens, NAC) and cortical territories, where the density of cortical innervation shows a decreasing rostro-caudal gradient (Berger et al. 1991).

Dopaminergic meso-cortical fibers to the pre-frontal cortex and limbic areas project through a key structure, the medial forebrain bundle (MFB) that passes through the lateral hypothalamus and the basal forebrain (Yeomans 1989). This pathway was initially described in humans with DTI and was shown to have two branches, the infero-medial branch (imMFB), typically following the lateral hypothalamus, and a superolateral branch (slMFB) realizing a connection of the VTA to reward-related subcortical and cortical regions (Coenen et al. 2009, 2011, 2012). This latter branch was further investigated in healthy subjects that underwent high-resolution anatomical magnetic resonance imaging including diffusion tensor imaging (Coenen et al. 2018). Using a global tractography approach, the slMFB and its connections to reward-related subcortical and cortical pre-frontal regions could be robustly tracked. The terminals are typically located in the dorso-lateral pre-frontal (DLPFC) and orbito-frontal cortices (OFC). The applied fiber selection algorithm is based on a seed region in a triangle between the SN, the red nucleus (RN) and the mamillo-thalamic tract—a “keyhole” region that ascending fibers of the VTA and medial SN have to pass on their way to subcortical (NAC, ALIC) and cortical target fields (Coenen et al. 2012).

The objective here is to describe those connections between VTA and sensory–motor cortex in humans, that harbor dopaminergic fibers and give rise to the dopaminergic terminals that were histologically detected throughout sensory–motor cortical fields. As the dopaminergic nature of fibers cannot be directly assessed as an inherent limitation of MRI technology, a constructive approach is the spatial orientation on the slMFB as a key structure that is known to conduct prominent dopaminergic afferents to pre-frontal cortex (Seamans and Yang 2004). As sensory and motor-related cortical areas are located caudally to pre-frontal regions which are addressed by slMFB connections, their dopaminergic projections are expected to be also caudally appended to the slMFB. To define these “motor extensions” of the MFB (i.e., the motorMFB), we apply a similar global tractography approach as in our previous study investigating the slMFB (Coenen et al. 2018). Based on branching/junction behavior, the motorMFB could be decomposed into three distinct sub-bundles. These bundles show a characteristic pattern of innervation among different cortical parcels defined by gyral anatomy and functional segregation into primary or secondary motor areas. The functional role of these bundles and dopaminergic innervation of motor areas is discussed.

## Materials and methods

We followed a two-step approach. The decision for seeds used to select the tracts of interest was guided by a normative connectome in MNI space. The selected seeds were then warped to subject space; streamlines were selected and rendered as fiber density maps. Subsequently, the fiber density maps were warped into MNI space, and averaged and analyzed. In the following, we detail out all processing steps.

### Subjects and magnetic resonance imaging

We used data from 100 subjects from the Human Connectome Project (Q1,S3) data corpus (resolution 1.25 mm isotropic, three b-shells with 1000, 2000, 3000; for more details on the protocol and preprocessing, see Glasser et al. (2013); age 29 ± 3.7, 36 males).

### Image processing

**Fig. 1:**
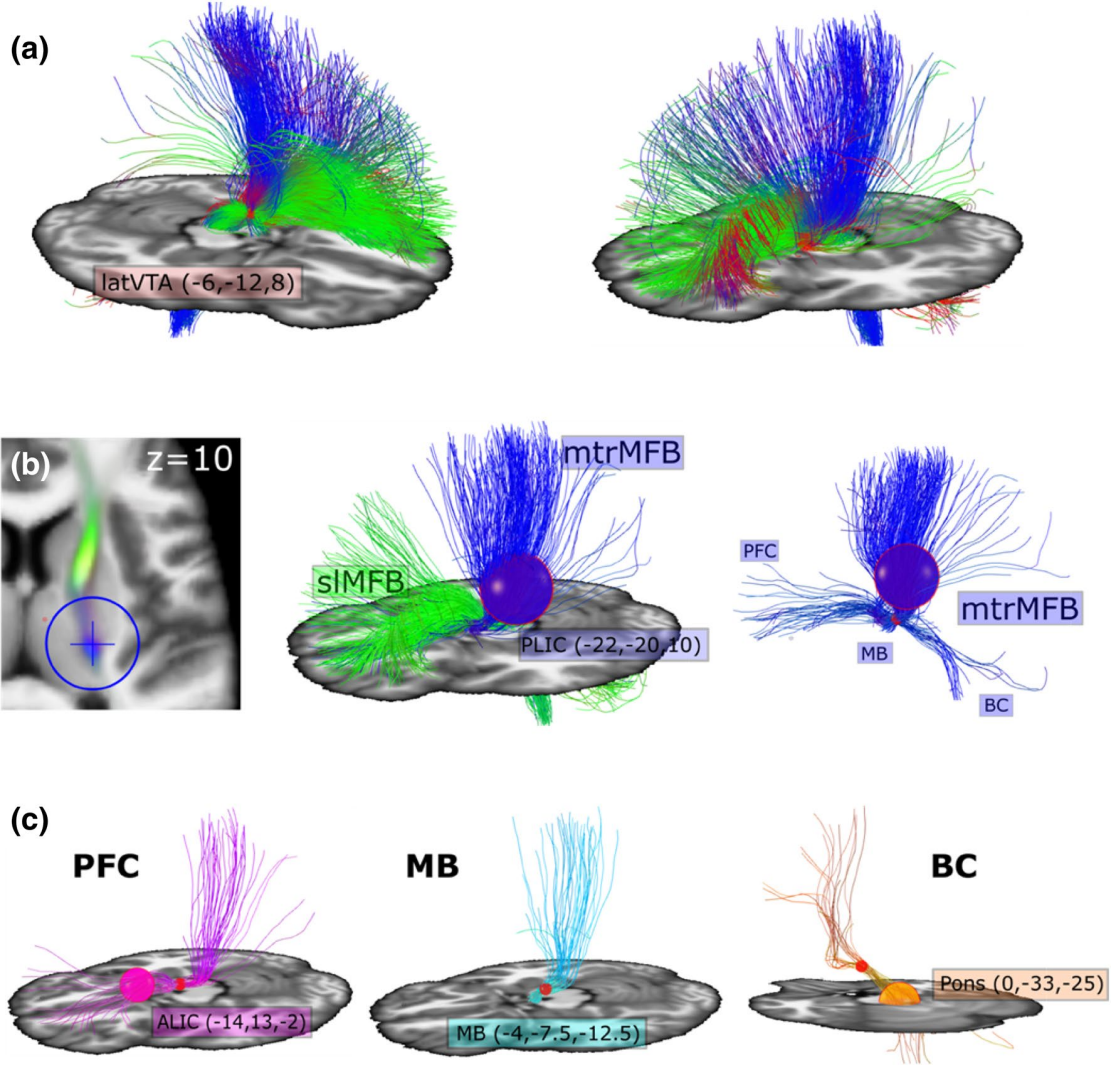
Fiber selection strategy–segregating the motorMFB into sub-bundles. **a** Indicates an unconstrained selection of fibers attached to the lateral VTA seed (*r* < 3 mm) initially described in Coenen et al. 2018. The lateral VTA seed (red) is located ventral from the red nucleus (magenta) and medial from the substantia nigra (yellow). In addition to the well known pre-frontal projections, numerous streamlines reach sensorimotor-related cortical areas. **b** Left: directional color-coded fiber density of the selection indicated in **a**. A clear bimodality is visible dividing the selection into a frontal/emotional part (slMFB, green) and a sensorimotor part (motorMFB, blue). Middle: a rather liberal seed (blue sphere; *r* < 15 mm) is set into the posterior limb of the internal capsule (PLIC) to capture the motorMFB. Right: the motorMFB is obviously composed of three distinct sub-bundles that can be defined by their origin: pre-frontal cortex (PFC), mammillary body (MB) and brainstem/cerebellum (BC). **c** Subdivision of the mtrMFB into three well-defined bundles selected by different seeds: an ALIC seed (magenta, *r* < 8 mm) targeting the PFC bundle; an MB seed (cyan, *r* < 3 mm) targeting the MB bundle; an Pons seed (orange; *r* < 10 mm) targeting the BC bundle. The red sphere indicates the latVTA seed (− 6, − 12, 8; *r* < 3 mm)

For selection of seeds, a normative connectome was constructed based on the considered subject cohort. Therefore, the raw diffusion data (dMRI) were warped to template space (MNI) and averaged over the group and tracked by the global tractography approach in Reisert et al. (2011, see details below). For normalization to MNI space, CAT12 (http://dbm.neuro.uni-jena.de/cat12/CAT12-Manual.pdf) using the Statistical Parametric Mapping software (SPM12, http://www.fil.ion.ucl.ac.uk/spm/software/spm12) was applied on the T1-weighted image. Then, the warp resulting from CAT12 was used for dMRI normalization. The reorientation of the raw dMRI data was based on the local Jacobian matrix (Raffelt et al. 2012) without modulation in angular domain. More precisely, for a certain voxel *x*, let *w*(*x*) be the displacement field, *J*(*x*) the corresponding Jacobian matrix. and *S*(*x*, *n*) the subject’s dMRI data for a certain *b* value (*n* is a direction on the b-shell). Then, the normalized data in MNI space take the form *S*′(*x*, *n*) = *S*(*w*(*x*), *J*(*x*) *n*). For interpolation in angular domain, an intermediate spherical harmonic representation was employed. The warping procedure was applied for each subject and the data were averaged in MNI space (on an isotropic grid of size 1.5 × 1.5 × 1.5 mm and 90 directions per b-shell). Once the template dMRI was constructed, the global tractography algorithm described in Reisert et al. (2011) with standard parameters was applied. A similar template connectome was also used in Coenen et al. (2018) for depiction of the slMFB. For further analysis, global tractography was performed in subject space and the seeds obtained from the considerations on the template connectome (Fig. 1) were warped to subject space for the selection of subject specific tracts.

### Tractography

White matter probability maps obtained from CAT12 were thresholded at a probability of 0.5 to determine the area of fiber reconstruction. For tractography, we followed the global approach (Reisert et al. 2011). As opposed to local walker-based tractography, global fiber tracking tries to find a fiber configuration that best explains the acquired diffusion weighted MRI data. Practically, the optimization process is similar to a polymerization process, where initially the streamlines are short and fuzzy, while during optimization connections, proliferate and fibers become more and more congruent with the data. The algorithm is based on the so-called “simulated annealing”. Initially, the system is at a rather high temperature, and the temperature is slowly decreased during iterations to obtain more and more accurate results. Usually, global fiber tractography is found to be less sensitive to noise and the fiber density is directly related to the measured data itself. We followed the algorithm proposed in Reisert et al. (2011). The provided toolbox contains two parameter sets; we have chosen the ‘dense’ preset. In addition, to increase the reproducibility, we increased the number of fibers by the following accumulation strategy: after the cooling down phase, the temperature was increased again to 0.1 and the state is further iterated for 10^7^ iterations. This procedure is iterated over five rounds and the tracts resulting from each round were accumulated to obtain one final tractogram which is five times larger than the initial one. This approach was proposed in Schumacher et al. (2018) and showed a much higher retest reliability.

### Reconstruction of the motorMFB

Tracking of the medial forebrain bundle has been previously performed by our group (Coenen et al. 2018). This approach is based on the definition of a seed that was applied to select relevant streamlines from the globally reconstructed connectome of the individual subjects. This seed was defined as a spherical region of radius 3 mm with center (latVTA = ± 6, − 12, − 8) in MNI space and is located within the triangle between red nucleus, subthalamic nucleus/substantia nigra, and mammillo-thalamic tract and sits just lateral of the VTA (latVTA seed; Fig. 1a). This choice of common MNI coordinates was the result of previous multiple tracking efforts with deterministic tractography (Coenen et al. 2009, 2011, 2012; Schlaepfer et al. 2013). The entire streamlines attached to the latVTA seed are displayed in Fig. 1a. To separate frontally projecting fibers of the slMFB that run in the anterior limb of the internal capsule (ALIC) from those projecting to motor-related cortical areas that project within the posterior limb (PLIC), we implemented a PLIC seed (± 22, − 20, 10; radius 15 mm; Fig. 1b). The resulting “motorMFB” consists of fibers attached to both the latVTA and the PLIC seed. This selection strategy was applied to all fibers returned from the global tractography algorithm. The MNI reference was obtained from the corresponding CAT12 segmentation.

### Differential trajectories to cortical motor representation

From our initial results, it became obvious that the resulting motorMFB was composed of three sub-bundles that join at the level of the latVTA seed (Fig. 1b): a bundle that project to pre-frontal areas through the anterior limb of the internal capsule (ALIC); a bundle that runs within the medial VTA and ends within the mammillary body (MB); a bundle that projects through the pons to cerebellum and the deeper brainstem. To transfer this segregation to our fiber selection algorithm, further seeds were implemented in addition to the latVTA and PLIC seeds: an ALIC seed (± 14, 13, − 2; radius 8 mm) that selects PFC-projecting streamlines (PFC bundle); an MB seed (± 4, − 7.5, − 12.5; radius 3 mm) that select streamlines that run within the VTA (MB bundle); an unpaired Pons seed (± 0, − 33, − 25; radius 10 mm) that covers the pontine tegmentum and selects streamlines connected to the brainstem and cerebellum (BC bundle; Fig. 1c). To compare the resulting sub-bundles of the motorMFB to a well-defined pathway that is known to lie in close proximity, we furthermore tracked the dentato-rubro-thalamic tract (DRT) based on the anatomic specifications given by Akram and colleagues (Akram et al. 2018). Thus, we used the dentate nucleus and surrounding cerebellar hemisphere (± 17, − 58, − 29; radius 8 mm; Dimitrova et al. 2002), the superior cerebellar peduncle (± 8.5, − 40, − 30; radius 4 mm; Kanaan et al. 2016), and the contralateral latero-ventral thalamic nucleus (VL; ± 18.5, − 16, 5; radius 5 mm; WFU PickAtlas, Maldjian et al. 2003) as seed regions for streamline selection.

### Rendering of fiber/terminal-density maps

For each subject, fiber density maps of the motorMFB, the sub-bundles, and the DRT were rendered at a resolution of 2.5 × 2.5 × 2.5 mm by trilinear interpolation. Then, the fiber density maps are normalized to MNI space (by the warps provided by CAT12) and thresholded at a value of 1 mm of streamline length per voxel, and group averages of the streamline indicator images were built. To understand the true extension of the full slMFB, the structure was overlaid onto a T1W template in MNI space (axial and coronal, Figs. 3, 4, 5, 6, 7). To visualize the cortical areas of projection, a similar approach is used. Fiber terminal maps are rendered on a grid of resolution 2.5 × 2.5 × 2.5 mm by trilinear interpolation in subject space, smoothed by a Gaussian of FWHM 3 mm, warped to MNI space, and directly averaged. For visualization, the cortical surface is colored according to the terminal density (Fig. 9).

### Defining cortical projection areas

**Fig. 2:**
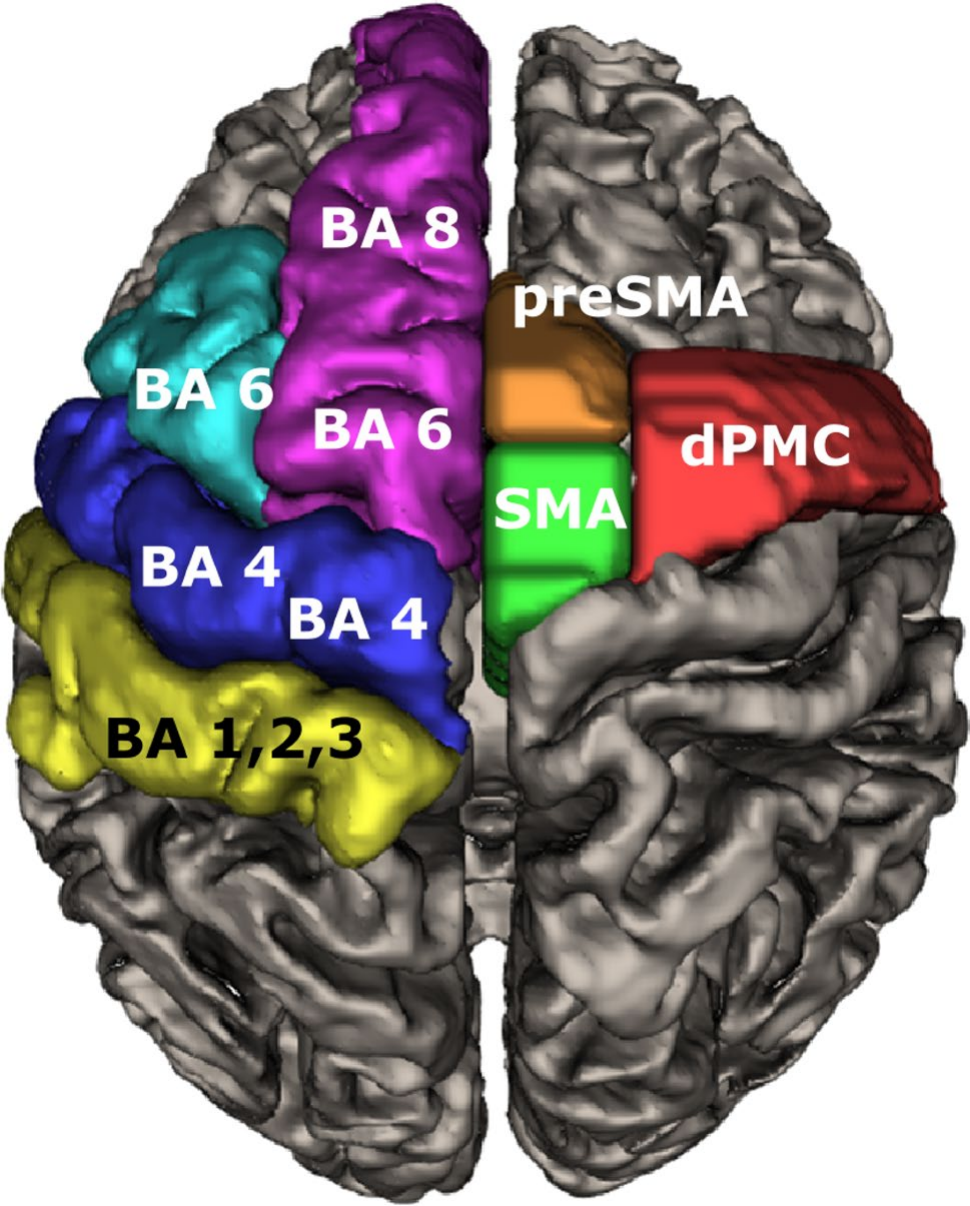
Topographical orientation. Left hemisphere indicates Brodmann area (Brodmann 1909) and cortical masks for cortex parcellation (Desikan/Killiany; Desikan et al. 2006) that were used to identify cortical projection fields. Magenta, superior-frontal gyrus; cyan, caudal middle-frontal gyrus; blue: precentral gyrus; yellow, postcentral gyrus. Right hemisphere indicates a functional subdivision of Brodmann area 6 based on a probabilistic mapping approach (Human Motor Area Template; Mayka et al. 2006). Green, supplementary motor area (SMA); orange, pre-supplementary motor area (pre-SMA); red, dorsal premotor cortex (dPMC)

To allocate the cortical projection fields of the motorMFB sub-bundles and the DRT, we referred to the cortical parcellation of the Desikan–Killiany atlas (Kanaan et al. 2016). The following cortical parcels were used: superior-frontal gyrus (magenta), caudal middle-frontal gyrus (cyan), precentral gyrus (blue), and postcentral gyrus (yellow; Fig. 2). To facilitate further discussion, parcells were compared to respective Brodmann regions (Brodmann 1909; Fig. 2). To display the functional subdivision of motor areas within the Brodmann area 6 (or the caudal middle-frontal and superior-frontal cortex respectively), we further referred to a probabilistic mapping approach using the Human Motor Area Template by courtesy of Professor D. Vaillancourt (Mayka et al. 2006). Thus, we could obtain cortical masks indicating the supplementary motor area (SMA; green), the pre-supplementary motor area (pre-SMA; orange), and the dorsal premotor cortex (dPMC; red; Fig. 2). As the ventral premotor cortex (vPMC) only receives a negligible amount of fibers, this region was not considered in further analysis. For quantitative analysis of streamlines reaching the cortical parcells, each of these ROIs was taken as an additional selection criterion for the terminals of selected streamlines from reconstructed bundles. As cortical parcells were rather flat, the terminal projections of our streamlines (the distal 20 mm) were tested for their presumed ending in the respective cortical ROI.

## Results

**Fig. 3:**
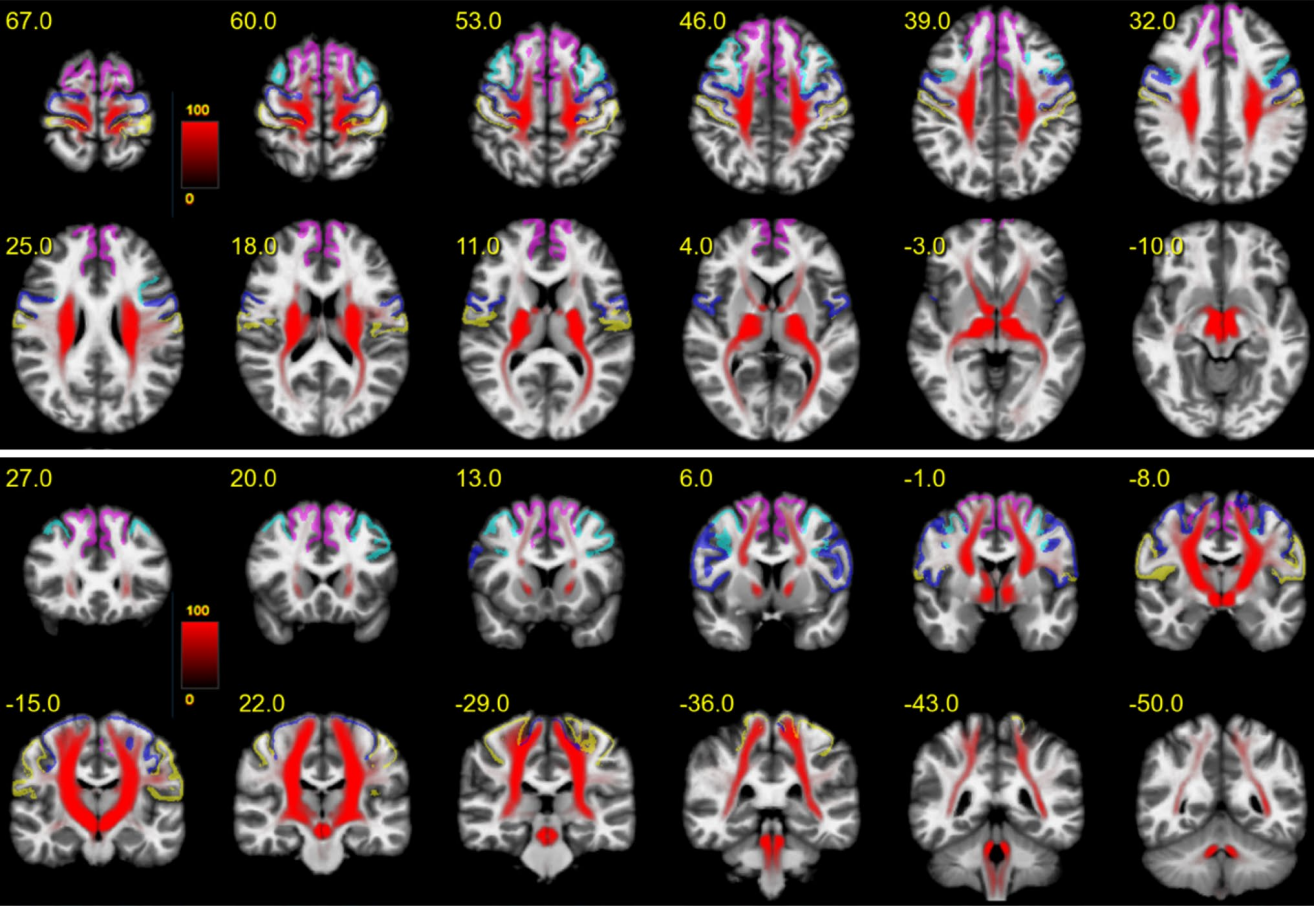
The entire motor MFB. Motor MFB (red) main trunk in MNI space (upper panel axial; lower panel coronal). Color coding in red indicates probability of occurrence of fiber streamlines in the entire group (in percent). Color-coding of cortical parcellation is analog to Fig. 2. A residual portion of streamlines was falsely attracted by the anterior commissure and the optic tract

Figure 3 shows an overview of the entire motor MFB (motorMFB) structure derived by global tracking in a probability map over all subjects in MNI space overlaid on a T1w template together with the idealized seeding region (upper panel axial and lower panel coronal). The vast majority of fibers project to sensory–motor cortical areas, and a residual portion of streamlines was falsely attracted by the anterior commissure and the optic tract.

**Fig. 4:**
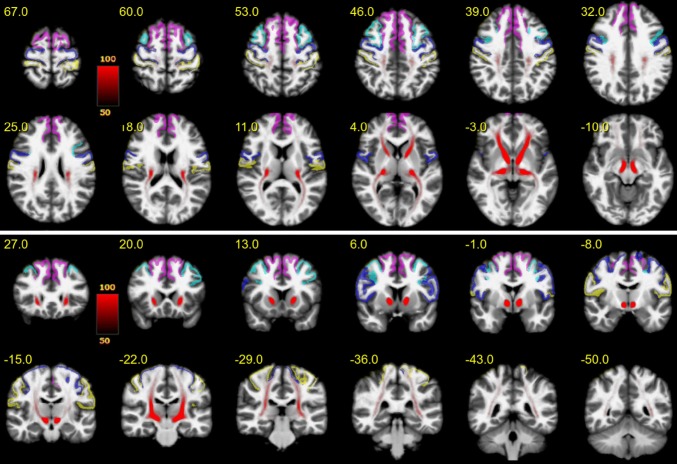
PFC bundle. Projections (red) connecting the pre-frontal cortex with sensory–motor areas in MNI space (upper panel axial; lower panel coronal). Color coding in red indicates probability of occurrence of fiber streamlines in the entire group (in percent). Color-coding of cortical parcellation is analog to Fig. 2

Figure 4 shows the PFC-sub-bundle derived by global tracking in a probability map over all subjects in MNI space overlaid on a T1w template together with the idealized seeding regions (upper panel axial and lower panel coronal). This bundle connects pre-frontal with primary sensory–motor cortical areas.

**Fig. 5:**
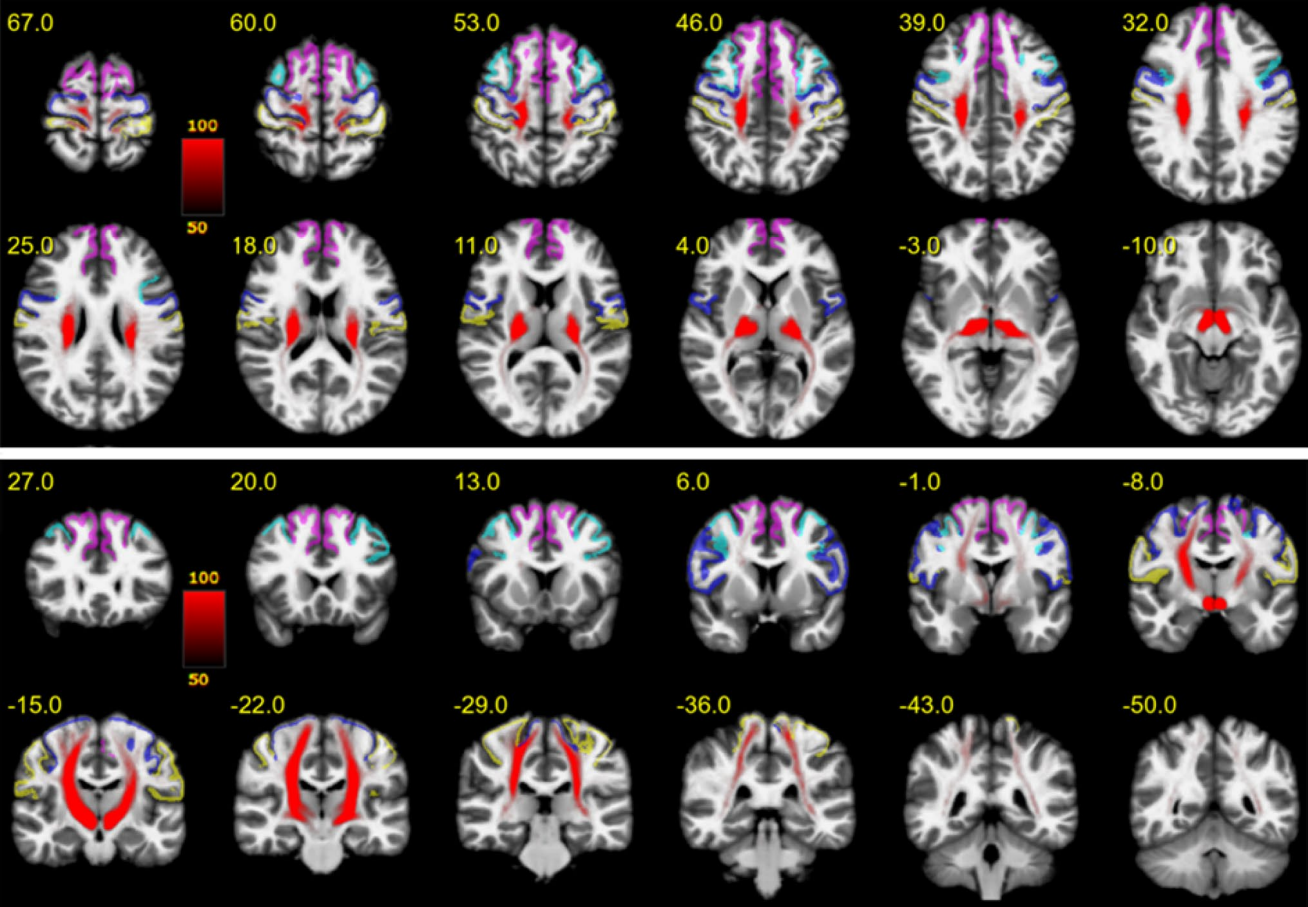
MB bundle. Projections originating within the mammillary bodies, passing the medial VTA and predominantly terminating within the pre-frontal cortex in MNI space (upper panel axial; lower panel coronal). Color coding in red indicates probability of occurrence of fiber streamlines in the entire group (in percent). Color-coding of cortical parcellation is analog to Fig. 1

Figure 5 shows the MB-sub-bundle derived by global tracking in a probability map over all subjects in MNI space overlaid on a T1w template together with the idealized seeding regions (upper panel axial and lower panel coronal). This bundle starts within the mammillary bodies, runs through the medial VTA and predominantly targets the primary motor cortex.

**Fig. 6:**
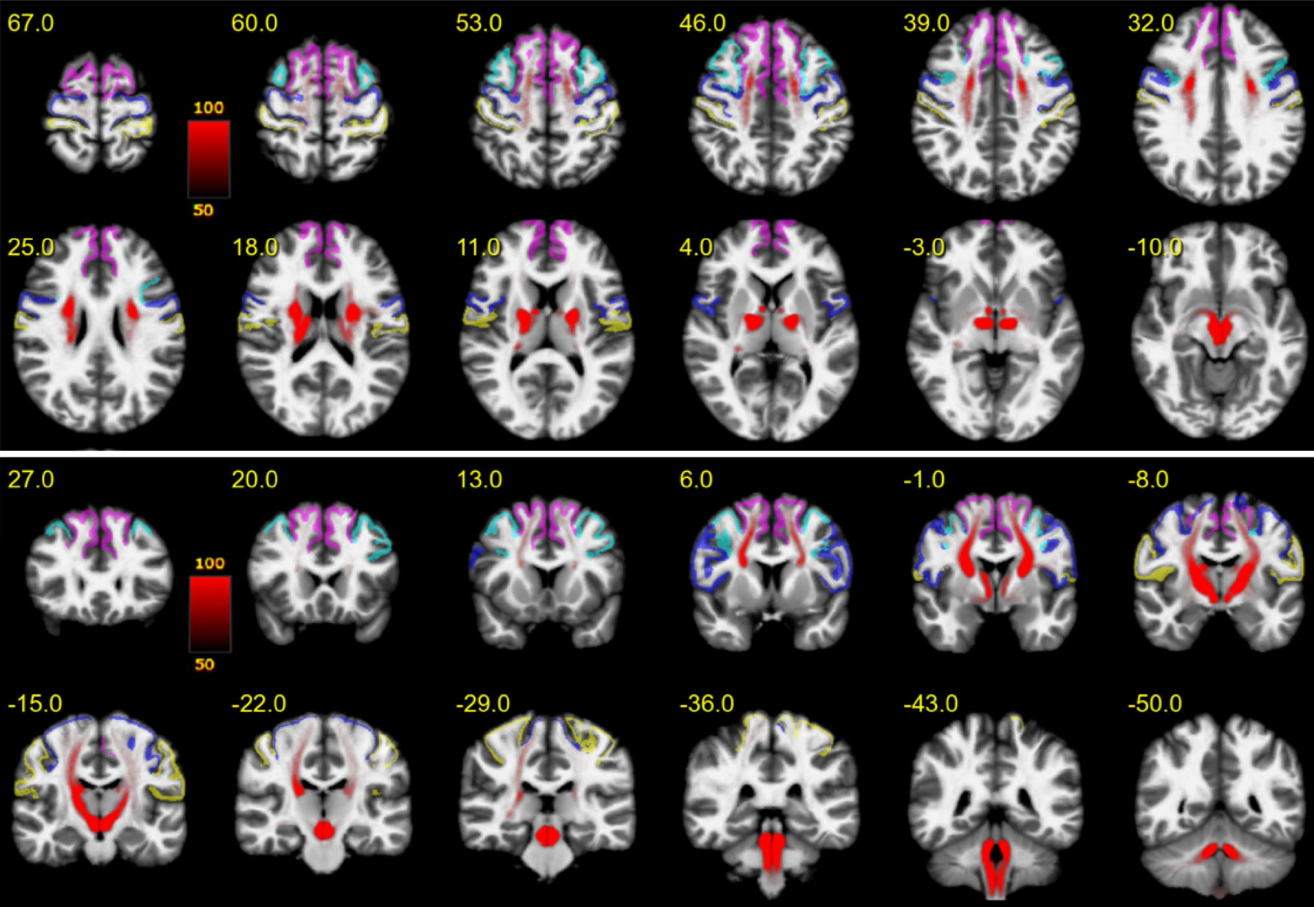
BC bundle. Projections originating within the brainstem or cerebellum mainly projecting towards the superior-frontal gyrus in MNI space (upper panel axial; lower panel coronal). Color coding in red indicates probability of occurrence of fiber streamlines in the entire group (in percent). Color-coding of cortical parcellation is analog to Fig. 1

Figure 6 shows the BC-sub-bundle derived by global tracking in a probability map over all subjects in MNI space overlaid on a T1w template together with the idealized seeding regions (upper panel axial and lower panel coronal). This bundle is characterized by a trunk connected to the ipsilateral brainstem and cerebellum. Fibers project mainly to the superior-frontal gyrus and to a lesser extend towards the caudal middle-frontal gyrus.

**Fig. 7:**
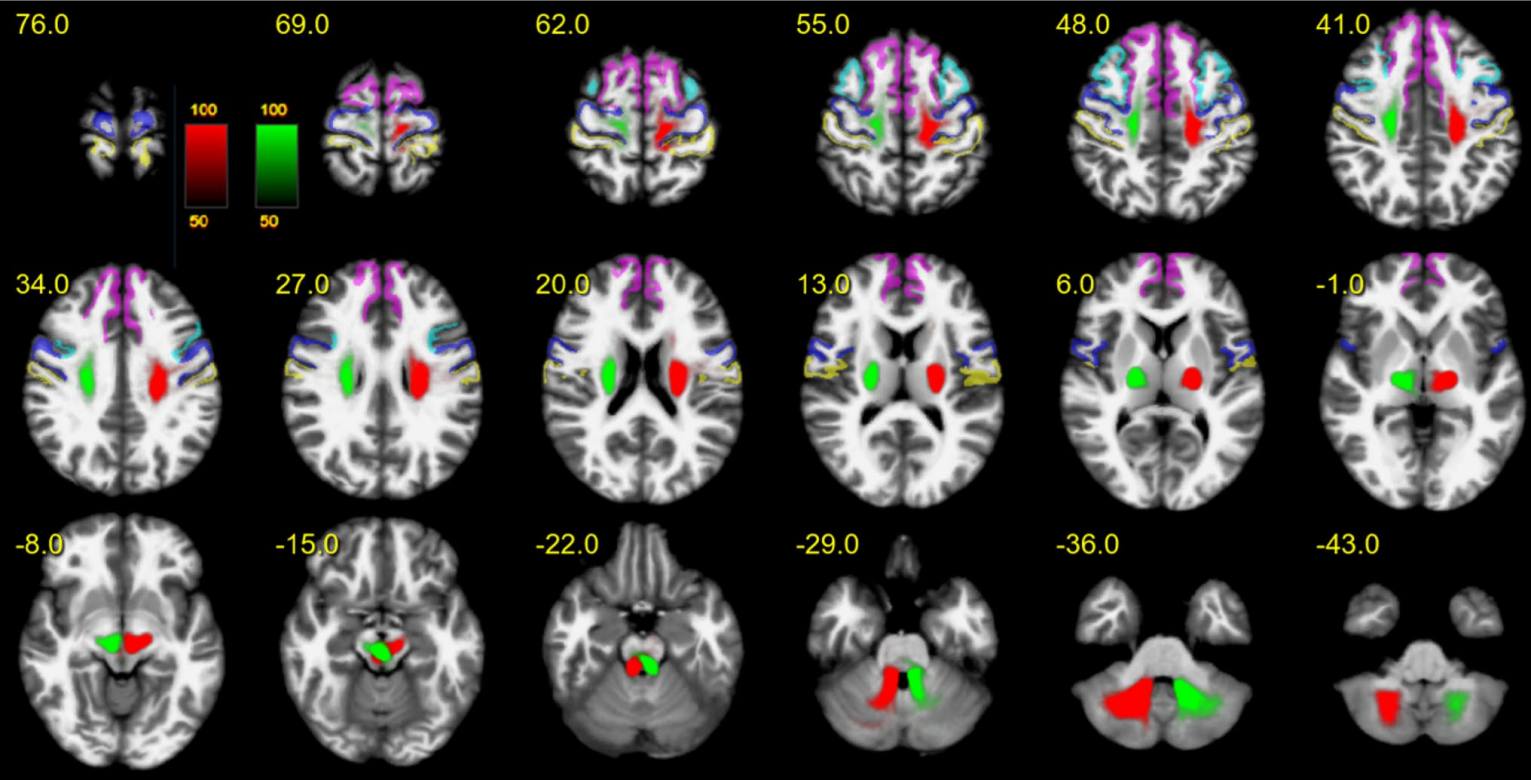
The dentato-rubro-thalamic tract (DRT). The dentato-rubro-thalamic tract (DRT) is in a close topographical and functional proximity with the bundles of the extended MFB. Fibers are indicated in MNI space (upper panel axial; lower panel coronal), for left/right side different colors were chosen to demonstrate the decussation on the pontomesencephalic level. Color coding in red or green indicates probability of occurrence of fiber streamlines in the entire group (in percent). Color coding of cortical parcellation is analog to Fig. 1

To methodologically validate the results of our tracking algorithm, we included a fiber tracking of the well-defined dentato-rubro-thalamic tract (DRT; Akram et al. 2018; Mollink et al. 2016). As this pathway is known to lie in close proximity with respect to fiber orientation and projection areas, it is, furthermore, an important control, excluding that DRT fibers were falsely attributed to the motorMFB. Tracking relied on a seed-based approach (seeds: dentate nucleus—superior cerebellar peduncle—contralateral ventro-lateral thalamic nucleus) analog to motorMFB sub-bundles. The result derived by global tracking in a probability map over all subjects is shown in Fig. 7. As expected, the tract is characterized by a decussation at the ponto-mesencephal level and a projection toward the superior-frontal and the precentral gyrus.

**Fig. 8:**
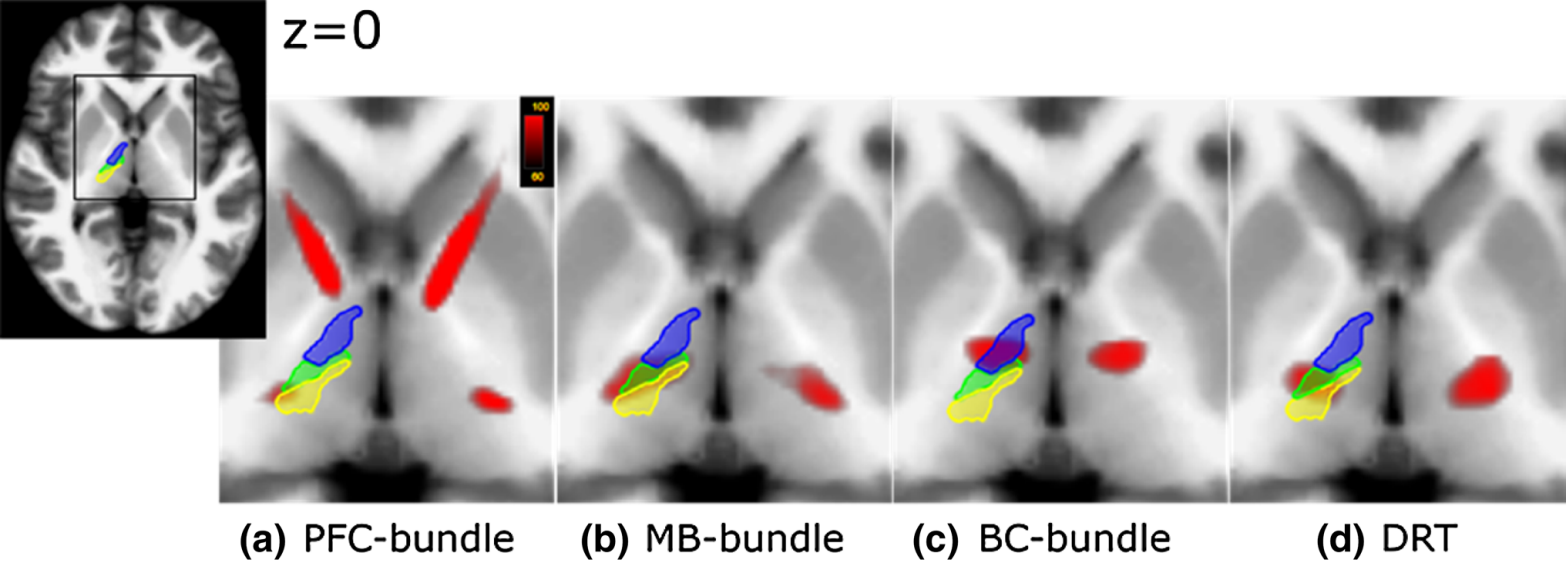
Reordering of bundles at the level of ventral thalamic nuclei. Magnification at the level of ventral thalamus (*z* = 0). Blue: ventral-anterior nucleus (VA); green: ventral-lateral nucleus (VL); yellow: ventral-posterior nucleus (VP). **a** The PFC bundle passes the ventral thalamus at the border zone between VP and VL. Color coding in red indicates probability of occurrence of fiber streamlines in the entire group (in percent, representative for **a**–**d**. **b** The MB bundle projects through the VL. **c** The BC bundle passes through the posterior zone of VA. **d** The DRT passes through the latero-posterior zone of the VL. The masks of ventral thalamic nuclei were adapted from Ilinsky et al. 2018

**Fig. 9:**
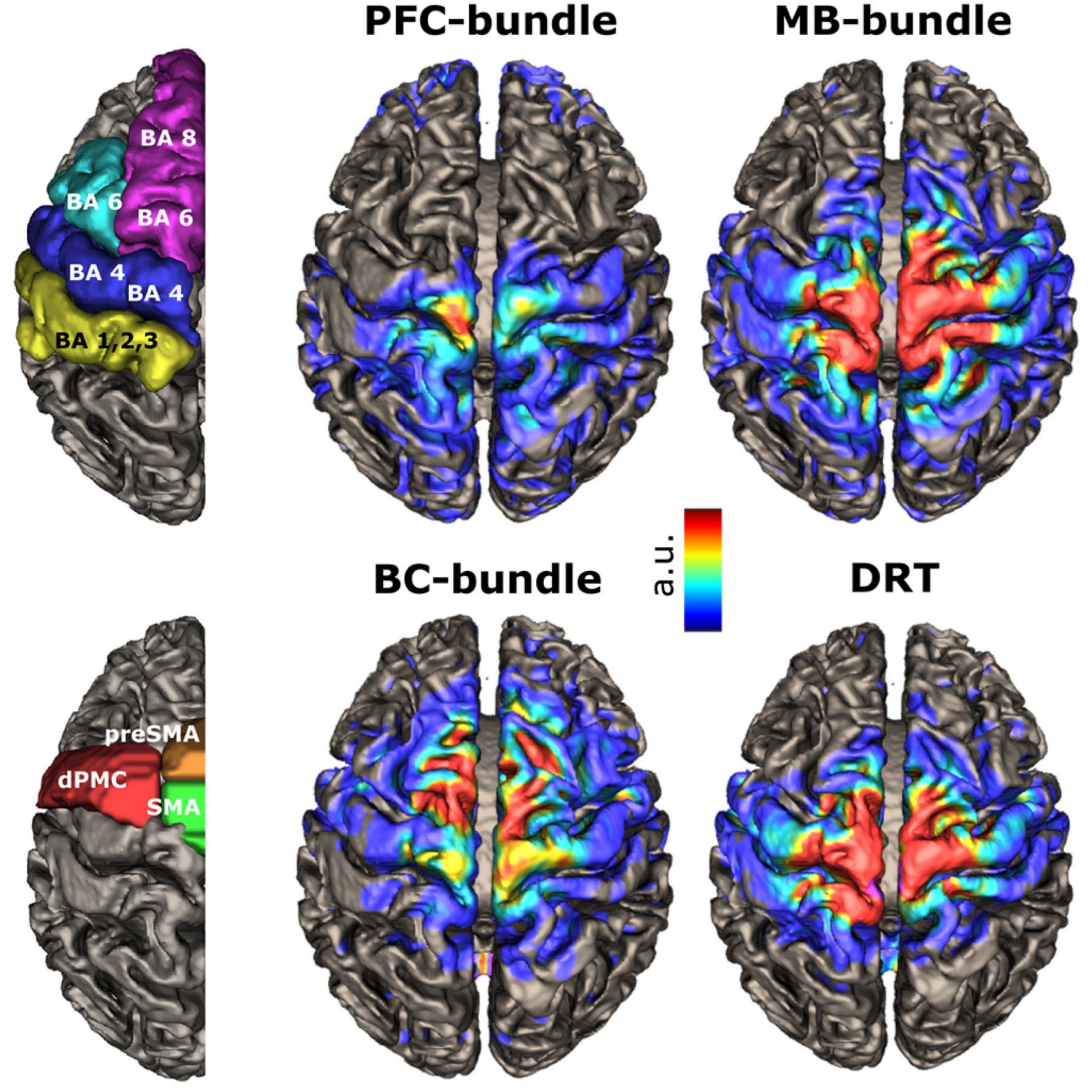
Cortical projection fields. The left panel indicates parcellation of sensory–motor cortices based on gyral anatomy, Brodmann area and probabilistic mapping as shown in Fig. 2. The right panel indicates the cortical projection fields of different motorMFB bundles and the DRT. Color coding indicates probability of occurrence of terminating fiber streamlines in the entire group (in arbitrary units)

At the level of ventral thalamic nuclei (i.e., ventral anterior, VA; ventral lateral, VL; and ventral-posterior thalamic nucleus, VP), a reordering of sub-bundles occurs (Fig. 8): the PFC bundle passes the thalamus at the border region of VL and VP, the MB bundle lies within the VL, whereas the BC bundle projects through the posterior VA. Similar to the MB bundle, the DRT passes through the VL nucleus. To capture the cortical projection patterns of motorMFB bundles and DRT, distribution of terminating streamlines was quantified within a cortical parcellation derived by Desikan and Killiany atlas (Desikan et al. 2006). To improve the functional segregation of motor cortical fields within the Brodmann area 6, masks derived from a probabilistic mapping approach (Human Motor Area Template; Mayka et al. 2006) were, furthermore, utilized. Figure 9 visualizes the cortical projection fields; percentage distribution is indicated in Table 1. According to their sequential arrangement at the level of ventral thalamic nuclei, motorMFB sub-bundles innervate the cortex in a rostro-caudal gradient. The BC bundle mainly projects to the superior-frontal and pre-frontal cortex thereby particularly covering the pre-SMA, dPMC, and SMA. The MB bundle mainly innervates the pre- and postcentral gyrus and also covers the SMA and dPMC. The PFC bundle shows the weakest cortical innervation that is restricted to the pre- and postcentral gyrus. In accordance to a similar fiber course within the VL (Fig. 8), the pattern of cortical innervation of the DRT is comparable to that of the MB bundle.

**Table 1:** Percentage distribution of cortical projections

	PFC bundle (%)	MB bundle (%)	BC bundle (%)	DRT (%)
Cortical region (Desikan/Killiany)				
Superior-frontal	18	12	30	17
Caudal middle-frontal	8	6	17	8
Precentral	31	57	31	59
Postcentral	22	23	10	16
BA 6 segmentation (HMAT)				
SMA	4	19	18	23
pre-SMA	1	6	15	6
dPMC	11	21	25	23

Percentages of fibers reaching specific subcortical WM targets are given relative to the whole projection of a particular bundle. Note, the percentages do not sum up to 100%, because the sub-bundles are not fully disjoint due to streamlines outside of the cortical parcels

## Discussion

As a consequence of the method, MRI-based fiber tracking bears a series of limitations: (1) it does not reveal the transmitter content of fibers; (2) it does not distinguish the directionality of projections and (3) it does not distinguish if a synaptic connection occurs or if the fiber simply passes through a structure. Thus, the exact fiber course of dopaminergic projections cannot be precisely determined.

### Why are dopaminergic projections required in motor cortical fields: data from rodents and non-human primates

The best functional characterization of dopaminergic innervation exists for the primary motor cortex (BA 4, pre-frontal cortex). In rats, M1 receives dopaminergic input from neurons in the ipsilateral VTA and adjacent medial SNC with a high target specificity (Hosp et al. 2015). A selective lesion of these neurons within the VTA induced an ipsilateral dopaminergic depletion within M1 and abolished motor learning with the contralateral paw, whereas execution of previously learned skills remained unchanged (Hosp et al. 2011a, b). In line with this finding, dopamine receptors and proteins involved in the regulation and intracellular signal transduction of these receptors become upregulated within M1 of the trained hemisphere in response to a reaching training (Hertler et al. 2016). Within M1, dopaminergic signaling promotes neuroplastic changes at multiple levels that are prerequisites for successful motor learning (for review, see Hosp and Luft 2013): (1) At the level of gene expression, dopamine induces cFos, a transcription factor that is expressed in M1 during skill acquisition (Hosp et al. 2011a, b). (2) At the level of synapses and synaptic plasticity, dopamine is required for the formation of long-term plasticity (LTP; Molina-Luna et al. 2009). Furthermore, dopamine critically regulates the balance between spine formation and elimination (Guo et al. 2015). (3) At the network level, dopamine strengthens motor representations and reduces cortical excitability (Hosp et al. 2009). In our present study, we found a direct connection between the VTA and M1 (i.e., the MB bundle). This bundle likely contains dopaminergic fibers and may be seen as the human analog to the VTA-M1 projection characterized in rats.

Compared to BA 4, there is only sparse information about the functional impact of dopaminergic signaling in BA 6. BA 6 can be further segregated into the supplementary motor area (SMA) and the premotor cortex (PMC). The SMA is involved in planning and temporal structuring of movements based on memorized information (Makoshi et al. 2011). Whereas the SMA proper is particularly engaged in externally generated movements, the pre-SMA is focused on the initiation of non-automatized internally generated movements (Schwartze et al. 2012). In contrast, the PMC is involved in the spatial and sensory guidance of movements and can be divided into a ventral and a dorsal portion (Hoshi and Tanji 2004). As fibers of the motor MFB only reach the dorsal part, we restricted our analysis to this area. The dPMC operates the preparation of guided reaching, e.g., by processing information about movement direction and the target to be reached (Graziano and Aflalo 2007).

In mice, axonal bouton formation in M2 (i.e., the rodent homologue of pre- and supplementary motor fields) is regulated by VTA activity and dopamine release (Mastwal et al. 2014) indicating an impact on synaptic plasticity. Injecting D1-receptor antagonists into the dPMC of primates during a delayed-reaching task, decreased activity of neurons involved into preparation of forelimb movements (Sawaguchi 1997). Knowledge of dopaminergic modulation of pre-SMA and SMA came from experiments in primates that were treated with 1-methyl-4-phenyl-1,2,3,6-tetrahydropyridine (MPTP), a toxin that destroys dopaminergic neurons. There, dopamine concentrations within the SMA and pre-SMA were significantly reduced (Elsworth et al. 1990) and neuronal activity during a delayed motor task significantly was decreased in both regions. Furthermore, cortical excitability of SMA was significantly reduced (Escola et al. 2003). MPTP exposition also interfered with beta-band modulation of local field potentials over SMA and pre-SMA associated with impaired predictive encoding of motor behavior during a visually cued reaching task (Hendrix et al. 2018). However, these results have to be interpreted with caution. As MPTP leads to a degeneration of dopaminergic neurons within the entire midbrain, alterations cannot be certainly attributed to a reduction of dopaminergic signaling within the cortex itself. As neurodegeneration also affects nigro-striatal dopaminergic projections, changes could be also induced due to an altered modulation of striatal neurons. With respect to BA 1,2,3, dense innervation of motorMFB fibers is restricted to the BA 3 subfield that is considered to be primary somatosensory cortex sensu stricto (Viaene et al. 2011). In rats, injection of D1- and D2-receptor antagonists induced an enlargement of somatosensory-evoked potentials (SEP)-amplitude consistent with an increased cortical excitability. Thus, by reducing S1 excitability, DA may serve focusing on relevant (= strong) somatosensory input, thereby improving signal-to-noise ratio and improving sensory discrimination (Hosp et al. 2011a, b).

In summary, dopamine exerts a mainly activating and facilitating effect on motor-related cortical networks. Within pre-SMA, SMA, and dPMC, dopaminergic signaling seems to support the temporal structuring and spatial guidance that is required for planning novel movement sequences. If finalized, dopamine promotes the storage of these motor engrams within M1 by supporting neuroplastic changes.

### Are there substantial inter-species differences with respect to the MFB or meso-cortical dopaminergic fibers?

The MFB cannot be defined as a tract that connects particular anatomical structures or that is characterized by a specific transmitter content. It is, furthermore, a complex compound of heterogeneous pathways that connects “lower” centers related to mood, motivation, and seeking (e.g., the VTA, the lateral hypothalamus or Nucleus accumbens) to “higher” limbic or cortical brain areas (Coenen et al. 2011, 2012). Within this connective highway, fibers containing all biogenic amine transmitters can be found. At the level of the VTA, the MFB becomes divided into two branches: the phylogenetic older infero-medial branch that reaches the lateral hypothalamus (i.e., the infero-medial bundle, imMFB) and a phylogenetic younger one, that runs through the anterior limb of the internal capsule and reaches, e.g., the pre-frontal cortex (i.e., the super-lateral bundle, slMFB; Coenen et al. 2009, 2012). Compared to rodents, the slMFB of humans is much more pronounced as a consequence of the phylogenetic development of the cortex. Interestingly, similar developmental changes have been described for the dopaminergic system (Berger et al. 1991; Puig et al. 2014): with progressing phylogenetic development of cortical fields, dopaminergic fibers within the meso-cortico-limbic system have grown in number and complexity, therefore accounting for the increased density of dopaminergic cortical innervation in humans compared to old-world monkeys or rodents (Berger et al. 1991; Raghanti et al. 2008). Similar to humans, dopaminergic fibers targeting the frontal cortex run within the MFB in rats (Aransay et al. 2015; Döbrössy et al. 2015) and in primates (Haber and Knutson 2010). Thus, between species, there is no substantial difference regarding function or principle anatomy of both the MFB and the dopaminergic meso-cortico-limbic system—but with respect to the complexity and magnitude of cortical innervation.

## How do dopaminergic meso-cortical projections reach the motor cortical fields?

Streamlines of the motorMFB can be prima vista segregated into three sub-bundles (Fig. 1): fibers coming from pre-frontal areas (PFC bundle), from the mammillary bodies (MB) and from brainstem and cerebellum (BC bundle). These bundles course through the ventral thalamic nuclei, thereby getting reordered (Fig. 8). PFC fibers pass the border zone between VL and VP nuclei, MB fibers run through the VL nucleus, whereas BC fibers cross the posterior portion of the VA nucleus. The ventral thalamic nuclei (particularly VA and VL) are considered to form the “motor thalamus (Mthal)” (Hamani et al. 2006) as this region is an interface between the motor areas of the cerebral cortex and motor-related subcortical structures, such as the cerebellum and basal ganglia (Bosch-Bouju et al. 2013). Although the knowledge of Mthal connections largely based on research in non-human primates, non-invasive MRI-based investigations in humans show that the organization of cortico-thalamic connections are similar between species (Behrens et al. 2003). Functionally, the Mthal is related to the complex cognitive and proprioceptive control of movement (Middleton and Strick 2000). Experimental lesion in primates induced a broad range of movement disturbances (Bornschlegl and Asanuma 1987) and an impairment of motor learning (Canavan et al. 1989). With respect to the further fiber course within the posterior limb of the internal capsule (PLIC), the thalamic topography of projections becomes preserved: projections to pre- or supplementary motor areas are located anterior within the PLIC, projections to sensory areas are located in a posterior position, whereas M1 projecting fibers are interposed in between. Thus, this topographical order is consistent with the fiber anatomy investigated in macaques (Fries et al. 1993).

In our study, the BC bundle is characterized by a trunk connected to the ipsilateral cerebellum and submesencephal brainstem and a projection focused on the superior-frontal and pre-frontal gyrus, including particularly the pre-SMA and dorsal PMC. Thus, this bundle links proprioceptive and cerebellar information to premotor regions of the cortex. This is in line with the knowledge of connectivity of VA nucleus (McFarland and Haber 2002; Garcia-Munoz and Arbuthnott 2015) and experimental lesions in this area that induce a cerebellar-like syndrome with ataxia and dysmetria (Bornschlegl and Asanuma 1987).

The PFC bundle mainly project to the pre- and postcentral gyrus, i.e., the primary motor- and somatosensory cortex (BA 4 and BA 1,2,3), in line with the known cortical connection of the posterior VL and VP nuclei (McFarland and Haber 2002; Fang et al. 2006). It is, furthermore ,characterized by a connection to the basal pre-frontal cortex. Thus, it forms an inter-lobar tract connecting fronto-orbital to pre- and postcentral cortex. A similar U-shaped connection has been previously described in a study using spherical deconvolution diffusion tractography and post-mortem dissections in humans as a part of a “frontal longitudinal system” (FLS) that connects movement planning and execution (motor cortex) with an overall goal directed strategy (pre-frontal cortex; Catani et al. 2012). Lesions within this system disturb executive functions or attention and working-memory processes (Grafman 2002; Stuss et al. 2002).

Finally, the MB bundle consists of fibers clearly originating within the mammillary bodies. However, there are no reports on direct connections between the mammillary bodies, the ventral thalamus, and motor cortical areas neither in rodents nor in primates or humans. Thus, an “overshoot” from streamlines within the medial VTA onto the mammillary bodies during the tractography could explain this unexpected finding. On the other hand, hypothalamic hamartomas that are located in close proximity to the mammillary bodies are known to cause gelastic seizures, a special form of motor epileptic manifestation (Parvizi et al. 2011). A direct pathway between the mammillary region and the motor cortex could provide a plausible explanation for this phenomenon. Apart from its origin within the mammillary bodies, the MB bundle runs within the medial VTA over a longer distance. This bundle represents a direct connection between the origin of the meso-cortico-limbic pathway and the primary motor cortex.

In summary, dopaminergic fibers of the meso-cortico-limbic system originating within the VTA may project to motor cortical fields via three pathways: (1) they get appended to cerebellar and proprioceptive afferents projecting to VA of the Mthal and continue to the rostral “cognitive” motor fields such as pre-SMA and dPMC (BC bundle). (2) They get appended to a U-shaped inter-lobar tract that is part of the frontal longitudinal system and connects the basal pre-frontal cortex with pre- and postcentral fields via the posterior VL of Mthal (PFC bundle). (3) They directly project from the medial VTA towards the caudal “executive” motor fields (i.e., SMA and primary motor cortex), thereby passing the ventral-lateral thalamic nucleus (MB bundle).

## Motor cortical physiology in patients suffering from Parkinson’s disease (PD)

Comparable to the MPTP model, degeneration of dopaminergic neurons is not confined to the meso-cortico-limbic system in PD. Dopaminergic neurons within the VTA and medial portions are less viable and degenerate late in the time-course of disease (Brichta and Greengard 2014). Thus, even though a 70% reduction of DA fibers within M1 and other frontal cortical areas occurs in PD (Gaspar et al. 1991), changes in motor cortical physiology cannot be unequivocally attributed to the dopaminergic depletion of cortex. Instead, dopamine loss within the striatum could contribute, for example, to the abnormal synchronization of M1 with basal ganglia that can be found in PD patients (Crowell et al. 2012). Furthermore, the occurrence of Lewy body pathology and reduction of gray matter of M1 (Burciu and Vaillancourt 2018) and SMA (Jubault et al. 2011) indicate the emergence of degenerative processes independent of dopamine loss. Thus, an exhaustive review on motor cortical pathology in PD is beyond the scope of this article. In brief, cortical activity during movements measured by changes in regional blood flow is increased in M1 and PMC, whereas a decrement in activation can be found in SMA (for review, see Lindenbach and Bishop 2013). With respect to motor cortical physiology, the capability to undergo plastic changes in response to paired associative stimulation or theta-burst stimulation protocols using transcranial magnetic stimulation (TMS) is extinguished in patients off dopaminergic medication (Lindenbach and Bishop 2013). Substitution of L-Dopa reinstated LTP/LTD-like plasticity in PD patients who had L-Dopa substitution for many years (Morgante et al. 2006; Ueki et al. 2006), whereas no or even disadvantageous effects were present in patients naive to l-Dopa (Kishore et al. 2012a, b). Thus, restoration of M1 plasticity likely is a long-term effect of l-DOPA treatment. In summary, capability for motor cortical plasticity formation in PD patients depends on duration of disease and persistent effects of L-Dopa treatment. In line with dysfunctional motor cortex plasticity, motor learning is impaired in PD patients (Frith et al. 1986; Verschueren et al. 1997). With respect to hypomania and increased impulsivity in Parkinson’s disease, a recent PET study found a hyperactivation in the right precentral gyrus, the right paracentral lobule, and the superior-frontal gyrus in hypomania (Schwartz et al. 2019), possibly allowing an interpretation into the direction of our results. Hypomania in subthalamic deep brain stimulation has, on the contrary, been attributed to stimulation of the anterior and medial STN or to a collateral stimulation of the slMFB (Coenen et al. 2009). A direct motor connection of VTA and M1 would make such a mechanism even more plausible.

### Dopaminergic innervation of sensorimotor cortical fields in neuropsychiatric disorders: schizophrenia and depression

In contrast to PD, there is only limited evidence on the role of sensorimotor dopaminergic innervation in neuropsychiatric diseases. For schizophrenia, an imbalance of over- (nigro-striatal system) and underactivity (meso-cortico-limbic system) is thought to contribute to the expression of positive and negative symptoms (Howes et al. 2017). With respect to the frontal cortex, a reduced dopamine release in response to amphetamine stimulation has been reported within the dorsal pre-frontal cortex (DLPFC) of patients suffering from schizophrenia (Slifstein et al. 2015). Furthermore, a decreased binding on D2-receptors has been detected within the anterior cingulate cortex (Suhara et al. 2002). This reduction on cortical dopaminergic transmission together with increased striatal dopamine levels may contribute to the aberrant coupling between cortical and subcortical motor areas (Walther 2015) leading to motor symptoms like catatonia, abnormal involuntary movements, and other motor dysfunctions (Strik et al. 2017). Furthermore, a reduction of motor cortical dopamine may explain motor-learning deficits that are present in patients suffering from schizophrenia (Midorikawa et al. 2008) and a reduced activation of M1 and S1 during the performance of a finger-tapping task (Singh et al. 2014).

With respect to depression, a downregulation of dopaminergic signaling is believed to be a major biological correlate for the symptom of anhedonia (Belujon and Grace 2017). Repetitive transcranial magnetic stimulation (TMS) of the dorsal frontal lobe has been successfully applied to improve the symptoms in depressed patients (Berman et al. 2000). In this setting, stimulation is thought to mainly influence the dorsal pre-frontal cortex (DLPFC; Lefaucheur et al. 2014). However, stimulation also affects M1 that it is located immediately posterior to the DLPFC, as a motor response can be elicited contralateral to the stimulated side depending on the pulse amplitude. In anesthetized monkeys, TMS over M1 induced a release of endogenous dopamine in the ventral striatum (Ohnishi et al. 2004), likely via activation of the meso-cortico-limbic pathway. In the light of our study, this effect could be explained by the presence of a direct anatomic connection between M1 and the ventral mesencephalon.

## Integrating dopaminergic motor projections into a holistic model

Roughly generalized, the meso-cortico-limbic system is thought to provide information about the value and significance of environmental stimuli to enable behavioral changes in response to these cues. Specific aspects like motivational value and saliency are represented by particular subgroups of dopaminergic neurons (Bromberg-Martin et al. 2010), whereas the time-course of dopamine release may encode if an external cue is rewarding or aversive (Schultz 2007). Within this construct, dopaminergic projections to the basolateral amygdala mediate affective and emotion-modulating components of external stimuli (Zarrindast and Khakpai 2015). Projections towards the pre-frontal cortex, however, influence attention selection (Chudasama and Robbins 2004) and working memory (Seamans and Yang 2004), thereby enabling an attentional shift towards a novel and potential challenging situation. Hippocampal dopaminergic projections to the hippocampus furthermore serve to establish and to stabilize hippocampus-dependent memories (Edelmann and Lessmann 2018) allowing to exploit past experience for future occasions (Broussard et al. 2016). Implementing the role of dopamine within motor-related cortical fields into this functional scheme of the meso-cortico-limbic system, one may hypothesize that salient, novel, or appetitive environmental stimuli facilitate the occurrence of plastic changes in motor cortical networks, thereby promoting motor learning. Thus, the holistic concept of a motor portion of the MFB links an attentional shift and an emotional reference to salient inputs with the ability to adapt motor reactions.

In addition to provide dopaminergic innervation to cortex, the fiber bundles defined in this study could conversely serve as leads for cortical efferents towards the VTA. To generate an appropriate output signal, comprehensive information is thought to be integrated within this structure (Haber and Fudge 1997; Schultz 2006; Oliva and Wanat 2016; Morales and Margolis 2017). For example, dopaminergic VTA neurons receive input from the pre-frontal cortex (Oliva and Wanat 2016; Morales and Margolis 2017) and electric stimulation of the PFC modulates their excitability (Lodge 2011). On the behavioral level, the PFC–VTA projection plays a crucial role in the control of addiction and inhibition of drug-seeking behavior (Koob and Volkow 2010). If and how sensory–motor input may modulate the activity of VTA neurons is not investigated yet. Interestingly, in a rodent model of skilled-reaching, M1-projecting dopaminergic VTA neurons were only activated by motor training and not by food reward alone (Leemburg et al. 2018). This motor specificity of activation may require a motor feedback to detect specific dopaminergic neurons that are coupled to particular cortical areas involved in motor-learning dependent plasticity.
